# Sex Differences in the Association of Fat and Inflammation Among People with Treated HIV Infection

**DOI:** 10.20411/pai.v4i1.304

**Published:** 2019-08-19

**Authors:** Marcelo Chen, Chung-Lieh Hung, Chun-Ho Yun, Allison R. Webel, Chris T. Longenecker

**Affiliations:** 1 Department of Urology; MacKay Memorial Hospital; Taipei, Taiwan; 2 Department of Cosmetic Applications and Management; Mackay Junior College of Medicine, Nursing and Management; Taipei, Taiwan; 3 Division of Cardiology; Department of Internal Medicine; Mackay Memorial Hospital; Taipei, Taiwan; 4 Department of Medicine; Mackay Medical College; Taipei, Taiwan; 5 Department of Radiology; Mackay Memorial Hospital; Taipei, Taiwan; 6 Department of Nursing; Mackay Junior College of Medicine; Nursing and Management; Taipei, Taiwan; 7 Frances Payne Bolton School of Nursing; Case Western Reserve University; Cleveland, Ohio; 8 Division of Cardiovascular Medicine; Case Western Reserve University School of Medicine; Cleveland, Ohio; 9 University Hospitals Harrington Heart & Vascular Institute; Cleveland, Ohio

**Keywords:** HIV, body mass index, adipose tissue, inflammation, computed tomography

## Abstract

**Introduction:**

Ectopic fat deposition may contribute to chronic inflammation in people with HIV (PWH). To provide information for future mechanistic studies of metabolic risk in this population, we sought to determine which fat measures relate more strongly to inflammation and whether the fat-inflammation relationship is modified by sex or HIV status.

**Methods:**

We conducted a cross-sectional study of 105 PWH and 20 age- and sex-matched HIV-negative controls. Interleukin-6 (IL-6) and high-sensitivity C reactive protein (hs-CRP) levels were measured from plasma. Pericardial fat (PCF) and thoracic periaortic adipose tissue (TAT) volumes and peri-right coronary artery (RCA), left atrium (LA) roof, and liver densities were measured from cardiac CT scans. Unadjusted and multivariate adjusted linear regression models were used to determine the relationship between ectopic fat measures and inflammation biomarkers.

**Results:**

Forty participants had BMI < 25, 33 had BMI 25 to 30, and 52 had BMI > 30. Systolic blood pressure and insulin resistance increased with BMI. Participants with higher BMI had a higher CD4+ count. In models adjusted for demographics, HIV status, and metabolic risk factors, BMI was positively associated with IL-6 and hs-CRP. Ectopic PCF and TAT volumes were positively associated with IL-6 and hs-CRP; however, these relationships were somewhat attenuated in adjusted models. LA roof (but not peri-RCA) fat radiodensity was inversely associated with hs-CRP in fully adjusted models, and the association with IL-6 was borderline statistically signifi-cant (*P* = 0.054). IL-6 was more strongly associated with BMI and LA roof density in women than in men (*P* for interaction = 0.05).

**Conclusions:**

Among PWH receiving antiretroviral therapy, higher BMI and excessive ectopic fat burden were associated with circulating markers of systemic inflammation. Because these measures appear to be more strongly related to inflammation among women than men, future clinical studies of metabolic risk and inflammation among PWH should include sex-stratified analyses.

## INTRODUCTION

With the advent of effective antiretroviral therapy (ART), people with HIV (PWH) are living longer [1]. PWH can experience body fat changes, which can be related to age, duration of HIV infection, and ART [2-7]. Body fat changes have also been associated with HIV-related systemic inflammation and immune activation [8, 9]. Together with lifestyle behaviors (eg, physical activity, dietary intake), these factors influence body mass and systemic metabolism, which in turn may impact cardiovascular health in PWH.

Being overweight and obese is highly prevalent among PWH worldwide and varies by sex [10, 11] and geographic region [12]. Obesity is characterized by excessive ectopic fat deposition, which is related to cardiovascular disease [13, 14]. Obese PWH receiving ART have high levels of chronic inflammation and immune activation despite viral suppression. Total body adiposity—and particularly the quantity and radiodensity of ectopic visceral fat deposition around the heart and in the liver—may contribute to chronic inflammation in PWH [15, 16].

In this study, we examined the association of BMI and ectopic visceral fat burden with systemic inflammation. We further explored whether these associations were different in PWH compared to uninfected persons or in women compared to men.

## METHODS

### Participants

This analysis was conducted using the participants from the Boosting Health by Changing Activity (BOBCAT) study [17]. The BOBCAT study (NCT02553291) was a 6-month randomized clinical trial conducted in Cleveland, Ohio that tested the effect of a 6-session, behavior change intervention on physical activity and diet intervention among PWH. The study procedures and effects of the intervention on physical activity and diet have been reported elsewhere [17], and in this analysis we examined the baseline associations among BMI, computed tomography (CT)-derived ectopic visceral fat burden and systemic inflammation among sedentary, adult PWH. The University Hospitals Cleveland Medical Center IRB approved the study, and all participants signed written informed consent.

Of the 107 PWH enrolled in the BOBCAT study, 2 participants did not have a baseline CT scan. Therefore, 105 participants were available for this analysis. All PWH were receiving stable ART and had HIV-1 viral loads of <400 copies/mL for at least 1 year before enrollment. Twenty HIV-negative comparators were recruited as part of a pilot sub-study. All study participants were adults over 18 years of age with a Framingham 20-year CVD risk score > 20% for females and > 30% for males. Clinical history was obtained by chart review, and a targeted physical exam was performed to determine body anthropometrics, including body mass index (BMI), waist circumference, and waist-to-hip ratio. A fasting serum blood draw was used to measure HbA1c, glucose, and insulin. Insulin resistance was calculated using the homeostatic model assessment of insulin resistance (HOMA-IR) equation [18]. Standard BMI categories were used: underweight or normal (BMI < 25), overweight (BMI 25-30), and obese (BMI > 30).

### Cardiac Computed Tomography

All participants underwent a gated non-contrast 64-slice multi-detector CT scan of the chest for coronary artery calcium scoring. Three-millimeter slices were obtained from the carina to the liver with prospective ECG gating at 60% of the R-R interval. Ectopic fat volumes were measured offline on a dedicated workstation (Aquarius 3D Workstation, TeraRecon, San Mateo, CA), and included pericardial fat (PCF) volume and thoracic periaortic adipose tissue (TAT) volume in milliliters [19]. In addition, regional variation in the radiodensity of PCF was measured as the mean Hounsfield unit (HU) of one 10 mm^2^ region of interest in (A) the right AV groove at the level of the proximal RCA (peri-RCA) and (B) the region above the left atrium (LA roof) [20]. Mean liver density was measured in HU as a continuous measure that closely reflects the degree of fatty infiltration [21]. Three ROIs of 1cm^2^ were obtained in the parenchyma of the right lobe of the liver, taking care to avoid vascular structures and hepatic cysts. The average of these 3 measurements was used for analysis. A liver density < 40 HU was used as a clinically accepted measure of moderate to severe steatosis.

### Biomarkers of Inflammation

Two circulating biomarkers of systemic inflammation were measured. Interleukin-6 (IL-6) was measured by ELISA (Quantikine HS ELISA, R&D Systems) and high sensitivity C-reactive protein (hs-CRP) was measured by immunoturbidimetry (Beckman AU5800).

### Statistics

Scatter plots and correlation were used to analyze the relationships between BMI, markers of cardiometabolic risk, HIV status, and the markers of systemic inflammation IL-6 and hs-CRP. Variables were examined for departures from normal distribution and log transformation was performed when appropriate. Pearson correlations were used for normally distributed variables and Spearman correlations for non-normally distributed variables. Unadjusted and multivariate adjusted linear regression models were used to determine the relationship between the fat measures and natural-log-transformed IL-6 and hs-CRP. These included an unadjusted model, a model adjusting for age, sex, race, and HIV status, and a fully-adjusted model further adjusting for current smoking, systolic blood pressure, and insulin resistance (HOMA-IR). The interactions between BMI and HIV status and BMI and sex were explored with scatter plots and by adding interaction terms to the fully adjusted models. Sex-interactions with ectopic fat measures were then explored in a similar fashion. A *P*-value of < 0.05 was considered statistically significant, and a *P*-value between 0.05 and 0.10 was considered borderline statistically significant.

## RESULTS

Characteristics of the study population are shown by HIV status and sex in Table 1. A Supplemental Table further characterizes the participant characteristics by BMI category. Overall, the mean age was 52 years, 37% were female and 42% had BMI >30. Women had higher mean BMI (35 vs 27, women vs men; *P* < 0.001), but lower waist-to-hip ratio (0.92 vs 0.96; *P* = 0.007). Systolic blood pressure (*P* = 0.038) and HOMA-IR (*P* < 0.001) increased across BMI categories, but there was no statistically significant difference in HbA1c levels. PWH with higher BMI had higher CD4+ counts (*P* = 0.010).

**Table 1.** Participant Characteristics Stratified by HIV Status and Sex

	HIV-positive		HIV-negative	
	Men	Women	Men	Women
	N = 67	N = 38	N = 12	N = 8
**Demographics**				
Age, years	53 ± 7.7	52 ± 6.8	52 ± 7.0	46 ± 5.4
Race, African American	58 (87%)	34 (89%)	11 (92%)	8 (100%)
**Cardiometabolic Risk**				
SBP, mmHg	126 ± 16	122 ± 16	131 ± 24	133 ± 19
DBP, mmHg	80 ± 9.7	80 ± 10	83 ± 9.5	82 ± 8.2
HbA1c, %	5.8 ± 1.2	5.6 ± 0.54	5.9 ± 1.3	6.3 ± 1.3
HOMA-IR, units	3.3 ± 2.7	4.7 ± 3.7	3.0 ± 3.0	6.3 ± 4.4
Total Cholesterol, mg/dL	166 ± 36	192 ± 37	165 ± 35	176 ± 23
Triglycerides, mg/dL	138 ± 91	134 ± 93	77 ± 33	131 ± 73
HDL mg/dL	53 ± 16	59 ± 20	54 ± 15	49 ± 10
Statin use	10 (15%)	9 (24%)	1 (8%)	0 (0%)
**HIV status**				
CD4+ Count	599 (426–794)	662 (410–1082)	n/a	n/a
HIV-1 RNA < 20 c/mL	54 (81%)	34 (89%)	n/a	n/a
**Inflammation**				
IL-6, pg/mL	2.2 (1.4–3.9)	2.9 (1.9–4.4)	2.2 (1.7–2.5)	3.9 (2.5–4.3)
hs-CRP, mg/L	1.3 (0.6–3.5)	2.0 (0.9–6.3)	1.1 (0.7–3.1)	5.5 (1.9–8.5)
**Body size**				
BMI	27 ± 5.6	34 ± 9.7	29 ± 4.9	38 ± 7.8
Waist, cm	96 ± 15	109 ± 20	100 ± 16	117 ± 11
Waist-to-Hip ratio	0.96 ± 0.08	0.91 ± 0.08	0.94 ± 0.09	0.93 ± 0.07
**Ectopic visceral fat**				
PCF, mL	84 ± 44	87 ± 40	73 ± 30	92 ± 25
TAT, mL	11 ± 6.7	9.5 ± 4.8	7.3 ± 1.8	11 ± 5.1
Peri-RCA, HU	−106 ± 22	−98 ± 26	−88 ± 23	−108 ± 28
LA Roof, HU	−67 ± 23	−70 ± 24	−64 ± 19	−72 ± 20
Liver, HU	61 ± 6.4	60 ± 6.7	61 ± 7.4	58 ± 6.0

Data presented as mean ± standard deviation for continuous variables and n (%) for categorical variables. Because of highly skewed distribution, CD4+, IL-6 and hs-CRP are presented as median (interquartile range).

SBP: systolic blood pressure, DBP: diastolic blood pressure, HbA1c: glycated hemoglobin, HOMA-IR: homeostatic model assessment of insulin resistance, HDL: high density lipoprotein, BMI: body mass index, PCF: pericardial fat, TAT: thoracic periaortic adipose tissue, Peri-RCA: peri-right coronary artery, LA Roof: left atrium roof.

As expected, BMI positively correlated with PCF (r = 0.531, *P* < 0.001) and TAT (r = 0.393, *P* < 0.001) and negatively with liver density (r = −0.293, *P* = 0.001). Of the 2 novel measures of fat radiodensity, only LA roof density was inversely correlated with BMI (r = −0.483, *P* < 0.001), while peri-RCA density was not (r = 0.087, *P* = 0.35).

Table 2 describes the correlation of inflammation markers and ectopic fat measures with markers of cardiometabolic risk. Systolic BP and insulin resistance were the markers most consistently correlated with inflammation and ectopic fat. IL-6 was positively correlated with markers of cardiometabolic risk; however, correlations with hs-CRP were weaker and not statistically significant (all *P* > 0.05). Similarly, LA roof radiodensity was inversely correlated with SBP and HOMA-IR, but peri-RCA radiodensity was not. Correlations between ectopic fat volumes (PCF and TAT) and cardiometabolic risk were generally in the opposite direction of correlations between measures of fat density and cardiometabolic risk.

**Table 2.**
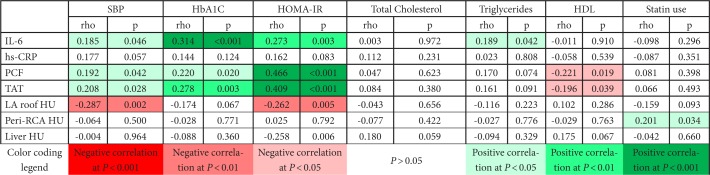
Correlation of Inflammation Markers and Ectopic Fat Measures with Markers of Cardiometabolic Risk

**Figure 1.**
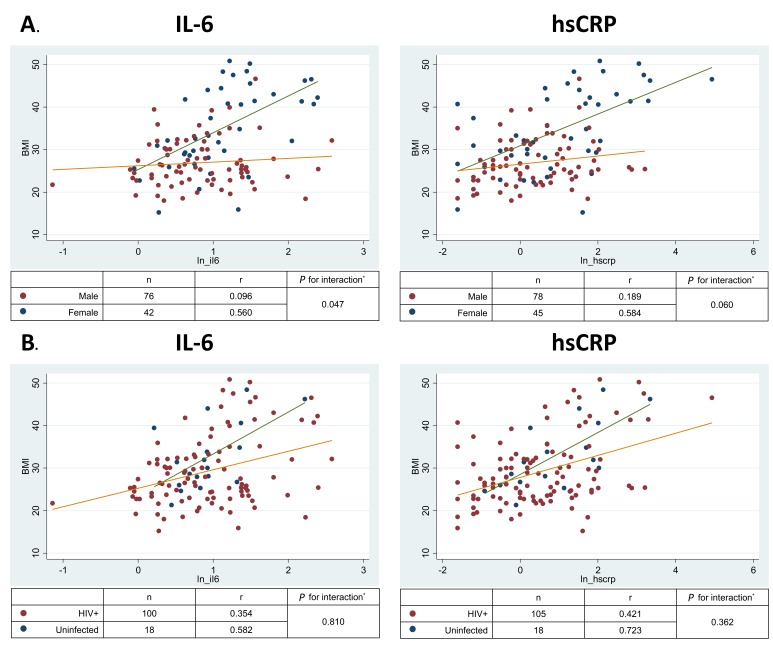
Relationship of BMI with biomarkers of inflammation by (A) sex and (B) HIV status. IL-6, Interleukin 6; hs-CRP, high sensitivity C reactive protein.

Table 3 displays the association of BMI and ectopic fat measures with (A) natural-log-transformed IL-6 and (B) natural-log-transformed hs-CRP values in unadjusted and adjusted linear regression models. In fully adjusted models, BMI was associated with both IL-6 (*P* = 0.001) and hs-CRP (*P* < 0.001). Scatter plots showed that BMI correlated with IL-6 (overall r = 0.308, *P* < 0.001) and hs-CRP concentrations (overall r = 0.389, *P* < 0.001); however, the strength of this association was significantly greater in women than in men (Figure 1A; for interaction in the fully adjusted model *P* = 0.047 for IL-6 and *P* = 0.06 for hs-CRP). In contrast, there was no significant interaction by HIV status (Figure 1B; *P* for interactions > 0.3).

**Table 3.** Association of BMI and Ectopic Fat Measures with Natural-Log-Transformed IL-6 and hs-CRP in Unadjusted and Adjusted Linear Regression Models

(A) Interleukin-6									
	Unadjusted			Adjusted for age, sex, race, HIV status			Fully adjusted^#^		
	β	95%CI	*P* value	β	95%CI	*P* value	β	95%CI	*P* value
**BMI**	0.029	0.016, 0.042	<0.001*	0.033	0.018, 0.048	<0.001*	0.031	0.013, 0.048	0.001*
**PCF**	0.004	0.001, 0.007	0.005*	0.004	0.001, 0.007	0.009*	0.003	−0.001, 0.006	0.111
**TAT**	0.020	−0.001, 0.040	0.054	0.018	−0.003, 0.038	0.086	0.005	−0.017, 0.028	0.641
**LA Roof**	−0.008	−0.013, −0.002	0.004*	−0.008	−0.013, −0.003	0.003*	−0.006	−0.011, 0.001	0.054
**Peri RCA**	−0.002	−0.007, 0.003	0.541	−0.001	−0.006, 0.004	0.561	−0.001	−0.006, 0.004	0.684
**Liver HU**	−0.005	−0.023, 0.013	0.579	−0.006	−0.025, 0.012	0.503	0.001	−0.019, 0.021	0.913

(B) Hs-CRP									
	Unadjusted			Adjusted for age, sex, race, HIV status			Fully adjusted^#^		
	β	95%CI	*P* value	β	95%CI	*P* value	β	95%CI	*P* value
**BMI**	0.074	0.049, 0.099	<0.001*	0.073	0.044, 0.103	<0.001*	0.075	0.041, 0.110	<0.001*
**PCF**	0.007	0.002, 0.013	0.010*	0.007	0.002, 0.013	0.012*	0.006	−0.001, 0.012	0.071
**TAT**	0.055	0.017, 0.093	0.005*	0.065	0.027, 0.104	0.001*	0.568	0.013, 0.101	0.012*
**LA Roof**	−0.017	−0.027, −0.008	<0.001*	−0.017	−0.027, −0.007	0.001*	−0.014	−0.026, −0.003	0.013*
**Peri RCA**	0.003	−0.007, 0.123	0.533	0.002	−0.008, 0.012	0.663	0.003	−0.007, 0.013	0.552
**Liver HU**	−0.013	−0.048, 0.022	0.469	−0.006	−0.043, 0.030	0.734	0.003	−0.036, 0.043	0.864

PCF: pericardial fat, TAT: thoracic periaortic adipose tissue, LA Roof: left atrium roof, Peri-RCA: peri-right coronary artery

# Fully adjusted model adjusts for age, sex, race, HIV status, insulin resistance (HOMA-IR), current smoking, and systolic blood pressure.

* *P* < 0.05

**Figure 2.**
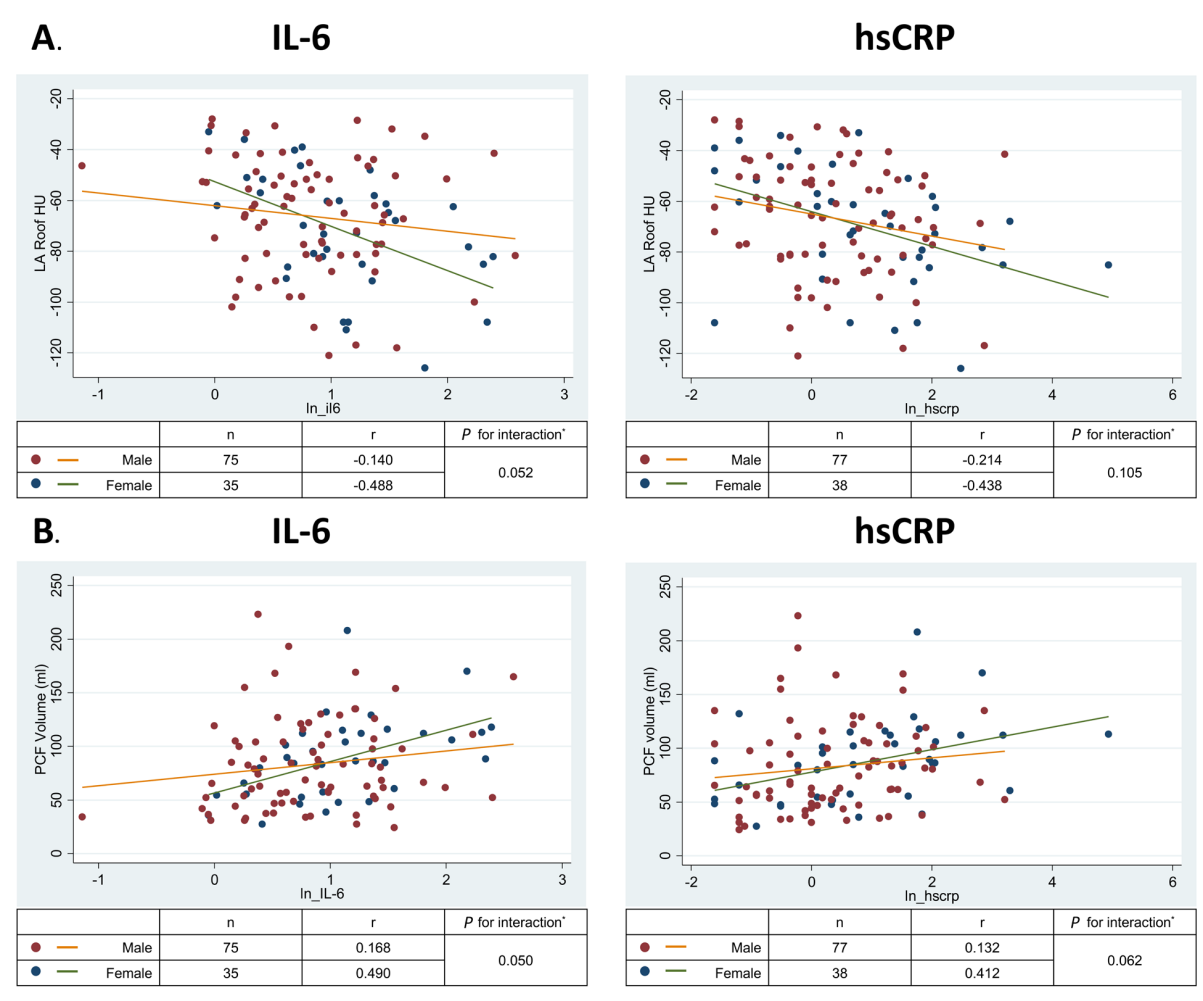
Relationship of left atrial roof pericardial fat density with interleukin-6 by sex.

Ectopic PCF and TAT volumes were positively associated with both IL-6 and hs-CRP (Table 3); however, adjustment for demographics and traditional risk factors somewhat attenuated these relationships. Furthermore, LA roof fat radiodensity (but not peri-RCA radiodensity) was inversely associated with hs-CRP in fully adjusted models and the association with IL-6 was borderline statistically significant (*P* = 0.054). In analyses of the sex-interaction, the magnitude of the inverse correlation between LA roof density and IL-6 and the positive correlation between PCF and IL-6 were stronger for women than for men (Figure 2A and 2B, interaction *P* = 0.05 for both). Similar interactions were observed for hs-CRP, but were statistically weaker (*P* = 0.06 to 0.11). Sex interactions with TAT and peri-RCA radiodensity for both markers of inflammation were not significant (all *P* > 0.1). Mean liver attenuation was not consistently associated with either IL-6 or hs-CRP (Table 3; all *P* > 0.4).

## DISCUSSION

Our findings demonstrate that among PWH receiving ART, total body adiposity and measures of ectopic fat volume and radiodensity are associated with circulating biomarkers of inflammation. We confirm and extend previous findings from our group [15, 16] by adding a measure of liver fat and regional assessment of PCF density in a new population of participants from a lifestyle intervention trial. Specifically, we show that total and ectopic fat burden appears to correlate with inflammation independently of traditional risk factors, and that the association of PCF density with inflammation may depend on the location of the measurement within the PCF fat depot. Importantly, we found evidence that the fat-inflammation relationship is stronger for women than men, but does not appear to differ by HIV status.

HIV infection and use of ART can lead to well-documented alterations in fat distribution and metabolic abnormalities [22]. The extent to which these pathological changes in fat structure and function are markers of or contribute in a causal way to chronic systemic inflammation and immune activation is an area of active research. If a phenotype of adiposopathy (ie “sick fat”) [23] can be identified among some with treated HIV infection using non-invasive imaging, then targeting these patients with lifestyle interventions and metabolic therapies may reduce the levels of chronic inflammation and immune activation that are thought to contribute to a wide range of non-AIDS chronic comorbidities such as cardiovascular disease and cancer.

An intervention that targets PCF specifically may have a greater impact on coronary heart disease, since it has been reported that PCF harbors proinflammatory cytokines that may directly influence the pathobiology of coronary atherosclerosis due to its close proximity and lack of fascial separation between the fat and arterial wall [24, 25]. We have previously shown that the PCF density is inversely associated with systemic inflammation [16]; however, in that study we measured global PCF density. The finding from this study that LA roof density is more strongly associated with systemic inflammation than peri-RCA fat may reflect regional differences in the association of PCF with the adiposopathy phenotype. This may represent differences in vascularity between a peri-coronary location and the LA roof [26]. Recent histologic studies [27] have confirmed that CT measured radiodensity of subcutaneous fat directly correlates with adiposity size among PWH receiving ART. Therefore, because pericardial fat is enriched with brown adipocytes that are smaller, more dense, and have high uncoupling protein-1 (UCP-1) expression [26], the pro-inflammatory adiposopathy phenotype may be reflected in the LA roof depot as a loss of UCP-1 activity, increased adipocyte size, and decreased PCF radiodensity. On the other hand, in the peri-RCA location, the correlation of radiodensity with adiposopathy may be confounded by local perivascular inflammation that increases radiodensity by increasing water content and fibrosis [20, 28].

Although sex and HIV status clearly affect fat distribution and function, whether the relationship between adiposity and inflammation differs by sex or HIV status is not clear [12, 29]. Many studies of PWH lack HIV-negative controls or sufficient numbers of female participants to evaluate these interactions. This appears to be the first study of PWH to show that the fat-inflammation relationship is stronger in women than in men. Our findings suggest that future observational studies and clinical trials of fat and inflammation in PWH should intentionally enroll enough women to allow for sex-stratified analyses. On the other hand, we did not find evidence that HIV status modifies the relationship between total body adiposity and inflammation. Although our study was nearly 40% female, the number of HIV-negative controls was small. Larger studies with more HIV-negative controls may be necessary to determine whether the fat-inflammation link is different in HIV-infected vs HIV-negative participants.

The other main limitation of this study is its cross-sectional nature, which may have resulted in residual confounding and does not allow for determination of causality. Additionally, we lacked detailed information on current individual ART drugs and drug classes. Because a recent clinical trial has suggested that differences in subcutaneous and visceral fat density change with different ART regimens [27], future studies should examine whether these differences are modified by sex. Finally, only a small proportion of participants met radiologic criteria for clinically significant hepatic steatosis, which may have limited our ability to detect relationships between liver fat and inflammation as has been reported by others [30].

## CONCLUSIONS

Among PWH receiving ART, higher BMI and excessive visceral fat burden were associated with circulating markers of systemic inflammation. Because these measures appear to be more strongly related to inflammation among women than men, future clinical studies of metabolic risk and inflammation among PWH should include sex-stratified analyses.

## SUPPLEMENTARY MATERIALS

**Supplementary Table.** Participant Characteristics Stratified by BMI

	BMI <25 n=40	BMI 25–30 n=33	BMI >25 n=52	*P* value (trend)
**Demographics**				
Age, years	54 ± 7	51 ± 8	51 ± 7	0.065
Sex, male	32 (80%)	24 (73%)	23 (44%)	<0.001*
Race, African American	38 (86%)	27 (82%)	48 (92%)	0.633
**Cardiometabolic risk**				
SBP, mmHg	122 ± 14	124 ± 15	129 ± 20	0.038*
DBP, mmHg	80 ± 11	80 ± 10	81 ± 9	0.368
HbA1c, %	5.7 ± 1.1	5.7 ± 1.2	5.9 ± 0.9	0.367
HOMA-IR, units	2.6 ± 2.2	3.0 ± 2.1	5.5 ± 3.8	<0.001*
Total Cholesterol, mg/dL	168 ± 33	174 ± 41	179 ± 38	0.192
Triglycerides, mg/dL	140 ± 111	127 ± 96	127 ± 58	0.500
HDL mg/dL	57 ± 19	54 ± 14	53 ± 17	0.275
Statin use				
**HIV status**				
HIV+	38 (95%)	26 (79%)	41 (79%)	0.058
CD4+ Count	563 ± 277	661 ± 335	809 ± 463	0.010*
**Inflammation**				
IL-6, pg/mL	2.6 ± 1.8	2.8 ± 2.0	3.8 ± 2.9	0.019*
hs-CRP, mg/L	1.8 ± 1.8	3.7 ± 5.7	7.8 ± 20.1	0.009*
**Body size**				
Waist, cm	83 ± 9	98 ± 9	117 ± 11	<0.001*
Waist-to-Hip ratio	0.91 ± 0.08	0.95 ± 0.07	0.96 ± 0.09	0.002*
**Ectopic visceral fat**				
PCF, mL	56 ± 23	79 ± 36	112 ± 37	<0.001*
TAT, mL	6.9 ± 3.3	9.3 ± 5.2	13.4 ± 6.4	<0.001*
Peri-RCA, HU	−104 ± 23	−105 ± 23	−99 ± 25	0.386
LA Roof, HU	−57 ± 19	−65 ± 21	−79 ± 22	<0.001*
Liver, HU	62 ± 6	63 ± 4	58 ± 7	0.009*

Data presented as mean ± standard deviation for continuous variables and n (%) for categorical variables.

SBP: systolic blood pressure, DBP: diastolic blood pressure, HbA1c: glycated hemoglobin, HOMA-IR: homeostatic model assessment of insulin resistance, HDL: high density lipoprotein, PCF: pericardial fat, TAT: thoracic periaortic adipose tissue, Peri-RCA: peri-right coronary artery, LA Roof: left atrium roof.

* *P*<0.05

## References

[1] Hogg RS, Heath KV, Yip B, Craib KJ, O'Shaughnessy MV, Schechter MT, Montaner JS. Improved survival among HIV-infected individuals following initiation of antiretroviral therapy. JAMA. 1998;279(6):450-4. PubMed PMID: 9466638. 9466638 10.1001/jama.279.6.450

[2] Addy CL, Gavrila A, Tsiodras S, Brodovicz K, Karchmer AW, Mantzoros CS. Hypoadiponectinemia is associated with insulin resistance, hypertriglyceridemia, and fat redistribution in human immunodeficiency virus-infected patients treated with highly active antiretroviral therapy. J Clin Endocrinol Metab. 2003;88(2):627-36. PubMed PMID: 12574192. doi: 10.1210/jc.2002-020795 12574192

[3] Caron M, Auclair M, Lagathu C, Lombes A, Walker UA, Kornprobst M, Capeau J. The HIV-1 nucleoside reverse transcriptase inhibitors stavudine and zidovudine alter adipocyte functions in vitro. AIDS. 2004;18(16):2127-36. PubMed PMID: 15577645. 15577645 10.1097/00002030-200411050-00004

[4] Carr A, Samaras K, Burton S, Law M, Freund J, Chisholm DJ, Cooper DA. A syndrome of peripheral lipodystrophy, hyperlipidaemia and insulin resistance in patients receiving HIV protease inhibitors. AIDS. 1998;12(7):F51-8. PubMed PMID: 9619798. 9619798 10.1097/00002030-199807000-00003

[5] Dube MP, Parker RA, Tebas P, Grinspoon SK, Zackin RA, Robbins GK, Roubenoff R, Shafer RW, Wininger DA, Meyer WA 3rd, Snyder SW, Mulligan K. Glucose metabolism, lipid, and body fat changes in antiretroviral-naive subjects randomized to nelfinavir or efavirenz plus dual nucleosides. AIDS. 2005;19(16):1807-18. PubMed PMID: 16227788. 16227788 10.1097/01.aids.0000183629.20041.bb

[6] Karmon SL, Moore RD, Dobs AS, Keruly J, Barnett S, Cofrancesco J Jr Body shape and composition in HIV-infected women: an urban cohort. HIV Med. 2005;6(4):245-52. PubMed PMID: 16011529. doi: 10.1111/j.1468-1293.2005.00284.x 16011529

[7] Saint-Marc T, Partisani M, Poizot-Martin I, Bruno F, Rouviere O, Lang JM, Gastaut JA, Touraine JL. A syndrome of peripheral fat wasting (lipodystrophy) in patients receiving long-term nucleoside analogue therapy. AIDS. 1999;13(13):1659-67. PubMed PMID: 10509567. 10509567 10.1097/00002030-199909100-00009

[8] Gallego-Escuredo JM, Villarroya J, Domingo P, Targarona EM, Alegre M, Domingo JC, Villarroya F, Giralt M. Differentially altered molecular signature of visceral adipose tissue in HIV-1-associated lipodystrophy. J Acquir Immune Defic Syndr. 2013;64(2):142-8. PubMed PMID: 23714743. doi: 10.1097/QAI.0b013e31829bdb67 23714743

[9] Grunfeld C, Pang M, Doerrler W, Shigenaga JK, Jensen P, Feingold KR. Lipids, lipo-proteins, triglyceride clearance, and cytokines in human immunodeficiency virus infection and the acquired immunodeficiency syndrome. J Clin Endocrinol Metab. 1992;74(5):1045-52. PubMed PMID: 1373735. doi: 10.1210/jcem.74.5.1373735 1373735

[10] Thompson-Paul AM, Wei SC, Mattson CL, Robertson M, Hernandez-Romieu AC, Bell TK, Skarbinski J. Obesity Among HIV-Infected Adults Receiving Medical Care in the United States: Data From the Cross-Sectional Medical Monitoring Project and National Health and Nutrition Examination Survey. Medicine (Baltimore). 2015;94(27):e1081 PubMed PMID: 26166086. Pubmed Central PMCID: PMC4504569. doi: 10.1097/MD.0000000000001081 26166086 PMC4504569

[11] Koethe JR, Jenkins CA, Lau B, Shepherd BE, Justice AC, Tate JP, Buchacz K, Napravnik S, Mayor AM, Horberg MA, Blashill AJ, Willig A, Wester CW, Silverberg MJ, Gill J, Thorne JE, Klein M, Eron JJ, Kitahata MM, Sterling TR, Moore RD, North American ACCoR, Design Rising Obesity Prevalence and Weight Gain Among Adults Starting Antiretroviral Therapy in the United States and Canada. AIDS research and human retroviruses. 2016;32(1):50-8. PubMed PMID: 26352511. Pubmed Central PMCID: PMC4692122. doi: 10.1089/aid.2015.0147 26352511 PMC4692122

[12] Godfrey C, Bremer A, Alba D, Apovian C, Koethe JR, Koliwad S, Lewis D, Lo J, Mc-Comsey GA, Eckard A, Srinivasa S, Trevillyan J, Palmer C, Grinspoon S. Obesity and Fat Metabolism in HIV-infected Individuals: Immunopathogenic Mechanisms and Clinical Implications. The Journal of infectious diseases. 2019 PubMed PMID: 30893434. doi: 10.1093/infdis/jiz118 PMC6941618 30893434

[13] Poirier P, Giles TD, Bray GA, Hong Y, Stern JS, Pi-Sunyer FX, Eckel RH, American Heart A, Obesity Committee of the Council on Nutrition PA, Metabolism Obesity and cardiovascular disease: pathophysiology, evaluation, and effect of weight loss: an update of the 1997 American Heart Association Scientific Statement on Obesity and Heart Disease from the Obesity Committee of the Council on Nutrition, Physical Activity, and Metabolism. Circulation. 2006;113(6):898-918. PubMed PMID: 16380542. 10.1161/CIRCULATIONAHA.106.171016 16380542

[14] Eckel RH, York DA, Rossner S, Hubbard V, Caterson I, St Jeor ST, Hayman LL, Mullis RM, Blair SN, American Heart A Prevention Conference VII: Obesity, a worldwide epidemic related to heart disease and stroke: executive summary. Circulation. 2004;110(18):2968-75. PubMed PMID: 15520336. doi: 10.1161/01.CIR.0000140086.88453.9A 15520336

[15] Longenecker CT, Jiang Y, Yun CH, Debanne S, Funderburg NT, Lederman MM, Storer N, Labbato DE, Bezerra HG, McComsey GA. Perivascular fat, inflammation, and cardiovascular risk in HIV-infected patients on antiretroviral therapy. Int J Cardiol. 2013;168(4):4039-45. PubMed PMID: 23886531. Pubmed Central PMCID: PMC3805774. doi: 10.1016/j.ijcard.2013.06.059 23886531 PMC3805774

[16] Longenecker CT, Margevicius S, Liu Y, Schluchter MD, Yun CH, Bezerra HG, Mc-Comsey GA. Effect of Pericardial Fat Volume and Density on Markers of Insulin Resistance and Inflammation in Patients With Human Immunodeficiency Virus Infection. The American journal of cardiology. 2017;120(8):1427-33. PubMed PMID: 28822563. Pubmed Central PMCID: PMC5614847. doi: 10.1016/j.amj-card.2017.07.019 28822563 PMC5614847

[17] Webel AR, Moore SM, Longenecker CT, Currie J, Horvat Davey C, Perazzo J, Sattar A, Josephson RA. Randomized Controlled Trial of the SystemCHANGE Intervention on Behaviors Related to Cardiovascular Risk in HIV+ Adults. Journal of acquired immune deficiency syndromes. 2018;78(1):23-33. PubMed PMID: 29373392. Pubmed Central PMCID: PMC5889354. doi: 10.1097/QAI.0000000000001635 29373392 PMC5889354

[18] Matthews DR, Hosker JP, Rudenski AS, Naylor BA, Treacher DF, Turner RC. Homeostasis model assessment: insulin resistance and beta-cell function from fasting plasma glucose and insulin concentrations in man. Diabetologia. 1985;28(7):412-9. PubMed PMID: 3899825. 3899825 10.1007/BF00280883

[19] Yun CH, Lin TY, Wu YJ, Liu CC, Kuo JY, Yeh HI, Yang FS, Chen SC, Hou CJ, Bezerra HG, Hung CL, Cury RC. Pericardial and thoracic peri-aortic adipose tissues contribute to systemic inflammation and calcified coronary atherosclerosis independent of body fat composition, anthropometric measures and traditional cardiovascular risks. European journal of radiology. 2012;81(4):749-56. PubMed PMID: 21334840. doi: 10.1016/j.ejrad.2011.01.035 21334840

[20] Konishi M, Sugiyama S, Sato Y, Oshima S, Sugamura K, Nozaki T, Ohba K, Matsubara J, Sumida H, Nagayoshi Y, Sakamoto K, Utsunomiya D, Awai K, Jinnouchi H, Matsuzawa Y, Yamashita Y, Asada Y, Kimura K, Umemura S, Ogawa H. Pericardial fat inflammation correlates with coronary artery disease. Atherosclerosis. 2010;213(2):649-55. PubMed PMID: 21040916. dio: 10.1016/j.atherosclerosis.2010.10.007 21040916

[21] Esterson YB, Grimaldi GM. Radiologic Imaging in Nonalcoholic Fatty Liver Disease and Nonalcoholic Steatohepatitis. Clin Liver Dis. 2018;22(1):93-108. PubMed PMID: 29128063. doi: 10.1016/j.cld.2017.08.005 29128063

[22] Buggey J, Longenecker CT. Heart fat in HIV: marker or mediator of risk? Current opinion in HIV and AIDS. 2017;12(6):572-8. PubMed PMID: 28796027. Pubmed Central PMCID: PMC5638646. doi: 10.1097/COH.0000000000000414 28796027 PMC5638646

[23] Bays HE. Adiposopathy is “sick fat” a cardiovascular disease? Journal of the American College of Cardiology. 2011;57(25):2461-73. PubMed PMID: 21679848. doi: 10.1016/j.jacc.2011.02.038 21679848

[24] Ding J, Hsu FC, Harris TB, Liu Y, Kritchevsky SB, Szklo M, Ouyang P, Espeland MA, Lohman KK, Criqui MH, Allison M, Bluemke DA, Carr JJ. The association of pericar-dial fat with incident coronary heart disease: the Multi-Ethnic Study of Atherosclerosis (MESA). Am J Clin Nutr. 2009;90(3):499-504. PubMed PMID: 19571212. Pubmed Central PMCID: PMC2728641. doi: 10.3945/ajcn.2008.27358 19571212 PMC2728641

[25] Mahabadi AA, Reinsch N, Lehmann N, Altenbernd J, Kalsch H, Seibel RM, Erbel R, Mohlenkamp S. Association of pericoronary fat volume with atherosclerotic plaque burden in the underlying coronary artery: a segment analysis. Atherosclerosis. 2010;211(1):195-9. PubMed PMID: 20223460. doi: 10.1016/j.atherosclerosis.2010.02.013 20223460

[26] Iacobellis G, Bianco AC. Epicardial adipose tissue: emerging physiological, patho-physiological and clinical features. Trends in endocrinology and metabolism: TEM. 2011;22(11):450-7. PubMed PMID: 21852149. doi: 10.1016/j.tem.2011.07.003 21852149 PMC4978122

[27] Lake JE, Moser C, Johnston L, Magyar C, Nelson SD, Erlandson KM, Brown TT, Mc-Comsey GA. CT Fat Density Accurately Reflects Histologic Fat Quality in Adults with HIV On and Off Antiretroviral Therapy. The Journal of clinical endocrinology and metabolism. 2019 PubMed PMID: 31329901. doi: 10.1210/jc.2018-02785 PMC6733493 31329901

[28] Antonopoulos AS, Sanna F, Sabharwal N, Thomas S, Oikonomou EK, Herdman L, Margaritis M, Shirodaria C, Kampoli A-M, Akoumianakis I, Petrou M, Sayeed R, Krasopoulos G, Psarros C, Ciccone P, Brophy CM, Digby J, Kelion A, Uberoi R, Anthony S, Alexopoulos N, Tousoulis D, Achenbach S, Neubauer S, Channon KM, Antoniades C. Detecting human coronary inflammation by imaging perivascular fat. Science Translational Medicine. 2017;9. 10.1126/scitranslmed.aal2658 28701474

[29] Varghese M, Griffin C, McKernan K, Eter L, Lanzetta N, Agarwal D, Abrishami S, Singer K. Sex Differences in Inflammatory Responses to Adipose Tissue Lipolysis in Diet-Induced Obesity. Endocrinology. 2019;160(2):293-312. PubMed PMID: 30544158. Pubmed Central PMCID: PMC6330175. doi: 10.1210/en.2018-00797 30544158 PMC6330175

[30] Price JC, Wang R, Seaberg EC, Budoff MJ, Kingsley LA, Palella FJ, Witt MD, Post WS, Thio CL. The Association of Inflammatory Markers With Nonalcoholic Fatty Liver Disease Differs by Human Immunodeficiency Virus Serostatus. Open Forum Infect Dis. 2017;4(3):ofx153 PubMed PMID: 28929125. Pubmed Central PMCID: PMC5601080. doi: 10.1093/ofid/ofx153 28929125 PMC5601080

